# Global Patterns of *Clostridioides difficile* Infection in Patients with Inflammatory Bowel Disease: A Systematic Review and Meta-Analysis of Prevalence, Epidemiology, and Risk Factors

**DOI:** 10.1093/crocol/otaf024

**Published:** 2025-03-27

**Authors:** Dominic Amakye, Paddy Ssentongo, Swapnil Patel, Shannon Dalessio, Smriti Kochhar, Arsh Momin, Kofi Clarke

**Affiliations:** Department of Medicine, Piedmont Athens Regional Medical Center, Athens, GA, United States; Division of Infectious Diseases, Department of Medicine, Penn State College of Medicine, Hershey, PA, United States; Department of Medicine, Penn State College of Medicine, Hershey, PA, United States; Division of Gastroenterology & Hepatology, Department of Medicine, Penn State College of Medicine, Hershey, PA, United States; Department of Medicine, Penn State College of Medicine, Hershey, PA, United States; Department of Medicine, Penn State College of Medicine, Hershey, PA, United States; Division of Gastroenterology & Hepatology, Department of Medicine, Penn State College of Medicine, Hershey, PA, United States

**Keywords:** *Clostridioides difficile* infection, inflammatory bowel disease, risk factors, epidemiology, meta-analysis

## Abstract

**Background:**

*Clostridioides difficile* infection (CDI) is a well-recognized complication of inflammatory bowel disease (IBD) that has been associated with poor clinical outcomes. The objective of this study is to characterize the global incidence, risk factors and outcomes of CDI in patients with IBD.

**Methods:**

A search of MEDLINE/PubMed, Scopus, and Cochrane Database of Systematic Reviews was conducted for studies published between January 1960, and March 2024. Random-effect models were employed to estimate the incidence of CDI in the IBD population. Risk factors and outcomes were estimated using random effects meta-regression and subgroup analysis.

**Results:**

Twenty-eight articles from 11 countries on 3 continents, comprising 796, 244 patients with IBD were included. The overall CDI rate was 8.84% (95% CI, 5.91%–13.03%). The rate of CDI was slightly higher in Asia at 11% (95% CI, 6.7%–18.44%) compared to the North America (USA and Canada) at 7.85% (95% CI, 3.80%–15.51%) and Europe, where the incidence, was 7.92% (95% CI, 3.87%–15.51%). A univariable random-effects meta-regression model demonstrated that male gender (odds ratio [OR], 1.18; 95% CI, 1.00–1.40) and older age (OR, 1.06; 95% CI, 0.99–1.15, per one-year increase in age) were factors associated with higher CDI incidence in the IBD population. CDI testing by PCR compared to enzyme immunoassay was associated with higher rates of CDI (OR, 4.70; 95% CI, 01.39–15.90). No association was observed between length of hospital stay and CDI.

**Conclusions:**

One in 10 patients with IBD were positive for CDI. Increasing age and male population were associated with higher risk of CDI.

## Graphical Abstract

**Graphical Abstract:**
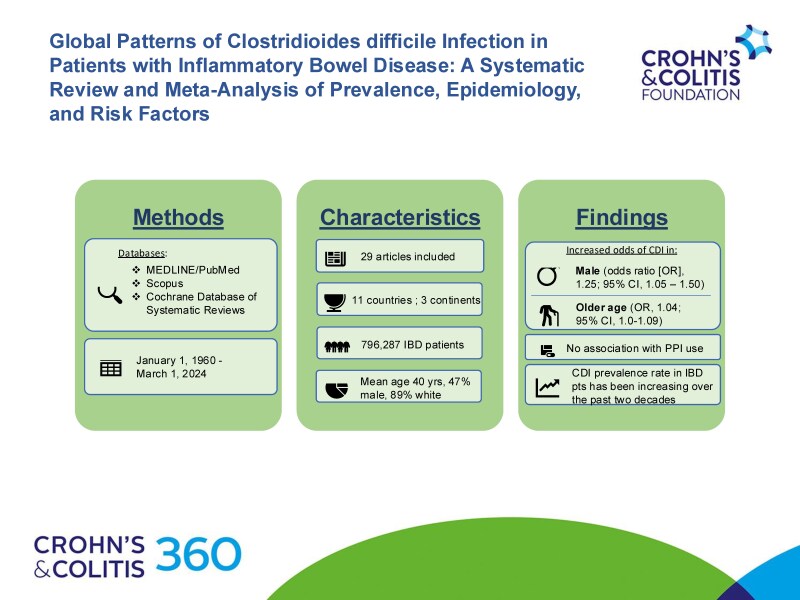

## Introduction

*Clostridioides difficile* (*C. diff*) infection (CDI) is caused by a gram-positive, fastidious, anaerobic, and spore-forming rod responsible for toxin-mediated infectious colitis, mainly associated with antibiotic use and healthcare interaction.^1^ It primarily affects the colon, leading to symptoms such as fever, diarrhea, and abdominal pain and often occurs after antibiotic use, which disrupts the normal gut microbiota, allowing *C. diff* to proliferate. The infection is characterized by inflammation of the colon, which can range from mild to severe and, in extreme cases, can lead to life-threatening conditions such as sepsis or toxic megacolon.^2^

Inflammatory bowel disease (IBD), encompassing Crohn’s disease (CD), and ulcerative colitis (UC), represent a complex spectrum of chronic inflammatory disorders affecting the gastrointestinal tract. The prevalence of IBD in the United States is estimated at 286 per 100,000 population for UC and at 201 per 100,000 for Crohn’s, which amounts to approximately 3 million Americans affected.^3^ Patients with IBD face numerous challenges beyond the primary symptoms of their condition, including an increased susceptibility to opportunistic infections due to immune dysregulation. They are more likely to be colonized with *C. diff* and develop active infection than the general population.^4^ Additionally the use of some long-term immunosuppressive therapies such as vedolizumab increases the risk of CDI.^5^ The prevalence of CDI carriage in patients with IBD is approximately 8 times higher than the non-IBD patient population.^6^

CDI in IBD patients has been associated with more severe disease flares, higher rates of hospitalization, longer hospital stay, increased severity of relapse, and increased risk of colectomy and mortality.^7–9^ This makes CDI a critical concern in the management of IBD. CDI incidence is rising among IBD patients.^10^

Treatment of CDI should be multifaceted. It should include treatment of the acute episode, evaluating and addressing any predisposing or risk factors and aiming to reduce recurrence. Effective options for antimicrobial therapy include metronidazole, oral vancomycin in various regimens, and fidaxomicin.^11^ Bezlotoximab, a monoclonal antibody that binds to toxin B, is used in conjunction with antibiotics to reduce risk of recurrence in high-risk patients. Fecal microbiota transplantation has been shown to be highly effective in cases of recurrent CDIs.^12^ Unfortunately, up to 50% of patients with IBD with CDI may require hospitalization and 205 may eventually go to surgery.^13^

There is a lack of systematic reviews and meta-analyses characterizing the epidemiology and risk factors of CDI in patients with IBD. Understanding the epidemiology and risk factors could aid in mitigating diagnostic challenges, thereby guiding optimal management strategies for CDI in the IBD population, which is paramount for improving clinical outcomes and reducing healthcare burden. This meta-analysis aims to characterize the global epidemiology, and the risk factors associated with CDI in IBD patients.

## Methods

### Data sources and extraction

We conducted a search of MEDLINE/PubMed, Scopus, and Cochrane Database of Systematic Reviews databases for studies published between January 1, 1960, and March 1, 2024, using a combination of medical subject headings and keywords in the title and abstract related to the incidence of *C. difficile* infection. We used terms “*C. difficile* infection” or *C. difficile* infection or *C. difficile* colitis or “CDI” combined with “Inflammatory bowel disease,” or “IBD” or “Crohn’s disease” OR “ulcerative colitis” along with keywords related to epidemiology (eg, incidence, prevalence, burden, mortality) to search for peer-reviewed publications. The search was performed for studies in all countries. This study followed the Meta-analysis of Observational Studies in Epidemiology (MOOSE) reporting guidelines and the Preferred Reporting Items for Systematic Reviews and Meta-analyses (PRISMA) reporting guidelines.^14,15^ For additional study material, we also searched the references cited by the retrieved articles. When narrowing the research studies, we applied the following inclusion criteria: (1) original peer-reviewed studies, and (2) identification of CDI through various screening methods (enzyme immune essay, molecular methods, and stool culture). Studies not conducted in humans, case reports, letters to the editor, case series, case-control studies, practice guidelines, meta-analyses, literature reviews, and commentaries were all excluded. We did not exclude studies based on the language of the article or country of origin.

CDI incidence in the IBD population was defined as the total number of patients with CDI divided by the total number of IBD patients tested for CDI in a defined population.

**Figure 1.**
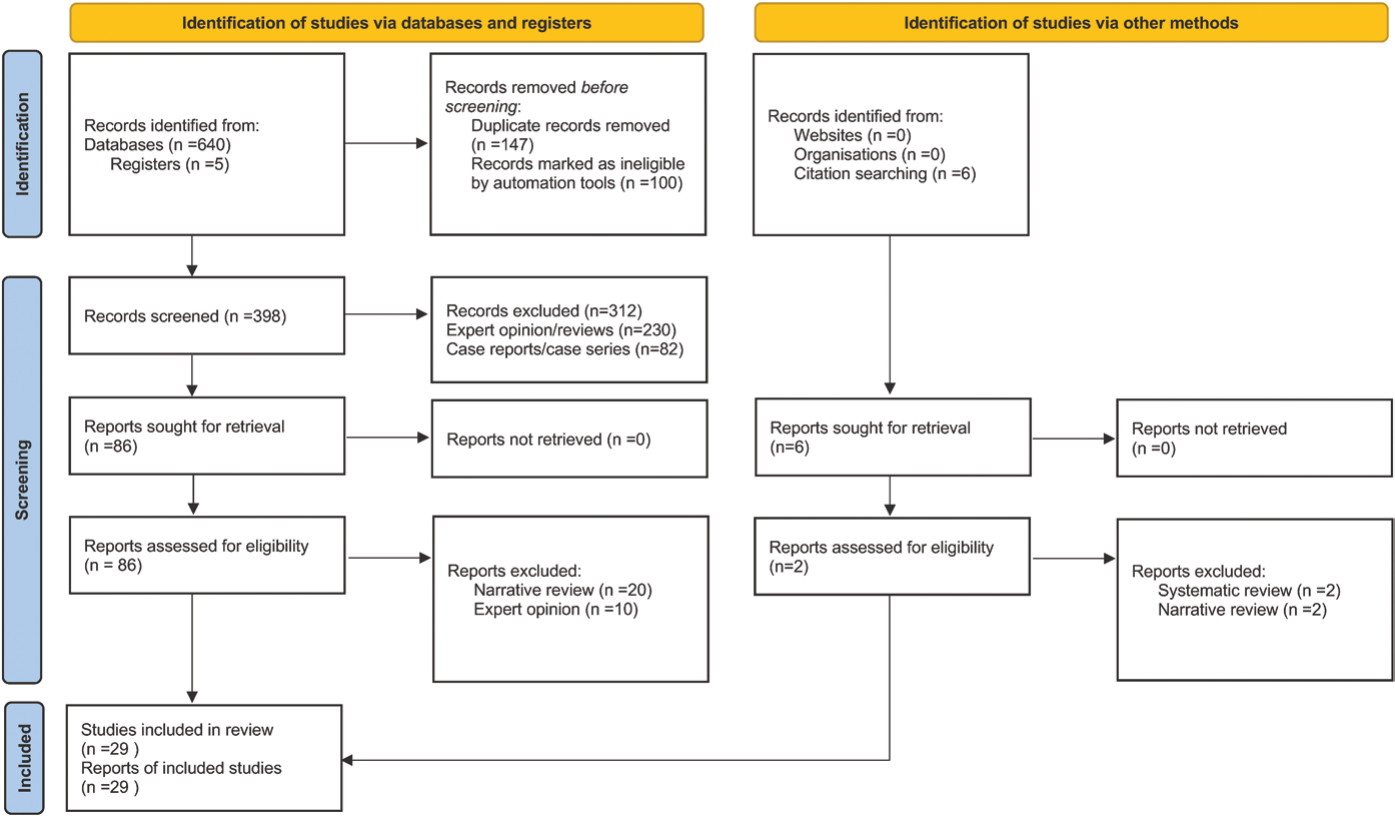
PRISMA flow diagram showing study selection.

We investigated each study and extracted detailed information on its characteristics and participants, including gender, race/ethnicity, mean or median age, methods used for CDI diagnosis, proportion exposed to antibiotics, length of hospital stay (LOS), proportion of IBD patients with UC or CD, mortality rate and the study’s methodologic quality. Three independent researchers (D.A., S.K, S.P.) initially independently screened the titles and abstracts of articles, obtained the full-text articles, and performed data extraction on those meeting the defined inclusion criteria. Then, all 3 researchers (D.A., S.K., and S.P.) jointly reviewed a random subset of articles to ensure selection accuracy. Disagreements about the included articles were resolved by one supervising researcher (P.S.). A detailed account of the inclusion/exclusion process is shown in PRISMA flow (**Figure 1**).

Two of the researchers (P.S. and A.M.) then independently assessed the quality of the articles included in the detailed analysis. Methodologic quality was assessed using the Newcastle-Ottawa Quality Assessment Scale, a validated tool for evaluating cross-sectional, case-control, and cohort studies.^16^ Scores of 8 to the maximum score of 9 were defined as high quality; scores of 5 to 7 were defined as intermediate quality, and scores of 1 to 4 were considered low quality. Studies were included regardless of the quality scores and risk of bias. Race or ethnicity was classified by the investigators of each study included in the meta-analysis, with categories selected defined by participants.

### Statistical Analysis

We adopted a narrative approach describing the number of studies, study settings, and diagnostic criteria for CDI. Descriptive statistics are reported as proportions of a population and as medians with interquartile ranges (IQR) in parentheses.

We applied random-effects models to estimate the incidence of CDI in the IBD population and their respective 95% confidence interval (CI), and we reported the pooled incidence. We used a generalized linear mixed-effects model with the logit link to pool the study estimates. We estimated all parameters via maximizing the pseudolikelihood. The generalized linear mixed-effects model method is not affected by the potential problems of back-transformation of Freeman-Tukey double arcsine transformation of single proportions.^17^ Individual and pooled estimates are displayed using forest plots. Between-study variation was assessed using *I*^2^, which describes the percentage of total variation across studies that is due to heterogeneity rather than chance, expressed as a percentage (low [25%], moderate [50%], and high [75%]).^18^ We report the pooled estimates as percentages.

We conducted a random effects meta-regression analysis to investigate the sources of heterogeneity. We examined the associations of each explanatory variable included in the meta-regression associated with the incidence of CDI. These variables included study-level median or mean age, the proportion of males, and the proportion of White individuals for studies conducted in the US or Canada.

We regressed the estimates as a function of the study year to explore the incidence trend.

To evaluate possible publication bias, we visually inspected the funnel plot for asymmetry by plotting the study effect size against SEs (standard error) of the effect size. We performed the Egger linear regression test and Begg rank correlation test.^19,20^ The Duval and Tweedie trim and fill procedure was used to adjust for the publication bias.^21^ An influence and outlier study sensitivity analysis was undertaken to estimate the association of each study with the overall pooled estimate.^22^ All statistical analyses were performed with R software, version 3.6.2 (R Foundation). From the R packages, the metaprop, escalc, and rma functions. The meta and metafor functions were used to conduct the analysis.^23^ The significance level was set at *P value *< 0.05, and all *P* values were 2-tailed.

## Results

### Characteristics of Studies

The initial literature search yielded 645 articles (**Figure 1**). After excluding duplicates, there were 398 unique studies. After a review of titles and abstracts, 312 articles which met exclusion criteria were excluded. We then comprehensively examined a total of 86 full-text articles and excluded 58 articles because they did not fulfill the inclusion criteria. Finally, a total of 28 articles were included in this meta-analysis. The final studies were from 11 countries on 3 continents. The present analysis included a total sample of 796 244 IBD patients. The number of patients included in individual studies ranged widely (minimum 54 to maximum 562 798), with a median of 120. Median age of study participants was 39 years (IQR: 36–43 y), 47% (IQR: 43–56 %) were males and 88% (IQR: 76–97 y) were Whites. The overall LOS was 7 days (IQR: 6–9). Details of each study included in the meta-analysis are provided in **Table 1**.

**Table 1.** Study characteristics.

Author	Year of publication	Country	Continent	Sample size	Male %	Mean age	CDI n	CDI incidence (%)	CDI diagnosis method	LOS all	PPI % all	CD_%	UC_%	Antibiotic cases %
Maharshak et al.^43^	2018	Israel	Asia	311	47	38.25	28	9.0	EIA	5	24	60.7 (58.6)	39.3 (39.4)	61
Meyer et a.l^45^	2004	USA	North America	54	46	49	10	18.51	EIA			55(36)11	33(44)20	90
Mylonaki et al.^46^	2004	UK	Europe	227	22	35	13	5.5	EIA			23.1	76.9	54
Ott et al.^47^	2011	Germany	Europe	521	20	24.5	10	1.9	Multiple			40	60	20
Sokol et al.^48^	2017	France	Europe	461	42	34.5	35	7.6	Culture		11	46.7(65.7)	53.3 (34.3)	20
Mundhra et al.^49^	2023	India	Asia	153	28	17.5	1	0.65	EIA				100	32
Vaishnavi et al.^50^	2003	India	Asia	94	26	NR	12	12.8	EIA				100	67
Iyer et al.^51^	2013	India	Asia	87	NR	40.2	3	3.5	EIA				100	67
Regnault et al.^8^	2014	France	Europe	483	55	32.5	34	7.0	Culture		11	58.5(57.8)	41.2(42.2)	35
Drozdinsky et al.^52^	2023	Israel	Asia	201	46	41	67	33.3	EIA	6		49.3	47.8	71
Li et al.^53^	2022	China	Asia	317	68	39	43	13.5	EIA, Culture	12	14	53.5(59.1)	46.5(37.6)	21
Varma et al.^54^	2020	USA	North America	102	46	NR	51	50	PCR	6.5		45(41)	55(59)	24
Gholam-Mostafaei et al.^55^	2019	Iran	Asia	140	NR	NR	24	17.14	PCR			8.3	91.6	NR
Shoaei et al.^56^	2019	Iran	Asia	85	60	NR	15	17.7	Multiple				100	100
Rezapour et al.^57^	2018	USA	North America	224500	NR	NR	3832	1.7	Multiple			77.8%	22.2	NR
Micic et al.^58^	2018	USA	North America	190	52	38	16	8.4	Multiple	Outpatient	15	72.1	27.9	6
Anderson et al.^59^	2017	USA	North America	198	43	45	66	33.33	PCR			60.1	39.9	91
Reinink et al.^60^	2017	USA	North America	117	49	44	27	23	EIA				100	26
Saffouri et al.^61^	2017	USA	North America	562798	47	NR	20798	3.7		9		20(34%)	80(65.9)	NR
Zhang et al.^62^	2016	China	Asia	646	64	NR	99	15.3	Multiple		35	40	60	43
Roy et al.^63^	2016	USA	North America	100	60	13	2	2				100		NR
Krishnarao et al.^64^	2015	USA	North America	137	51	NR	7	5	Multiple			57	42.9	54
Negron et al.^65^	2014	Canada	North America	278	56	NR	17	6.1	EIA			7.6	92.4	NR
Ananthakrishnan et al.^66^	2014	USA	North America	3188	39	55	35	1.1	EIA		48	49(55)	52(45)	95
Ananthakrishnan et al.^67^	2013	USA	North America	319	47	43	29	9.12	EIA				100	NR
Gros et al.^68^	2022	Spain	Europe	339	60	41	35	10.3	Multiple		8.8		100	9
Abdehagh et al.^69^	2021	Iran	Asia	120	44	36	17	14.2	Multiple			6	94	NR
Stoica et al.^70^	2015	Romania	Europe	78	63	NR	26	33.3	EIA			19.2(57.7)	80.8(42.3)	NR

Abbreviations: PCR: polymerase chain reaction, LOS: Length of hospital stay, means/ media; EIA: Enzyme immunoassay, USA: United States of America; NR: Not reported; (); Numbers in paracentesis are the controls for the studies.

**Figure 2.**
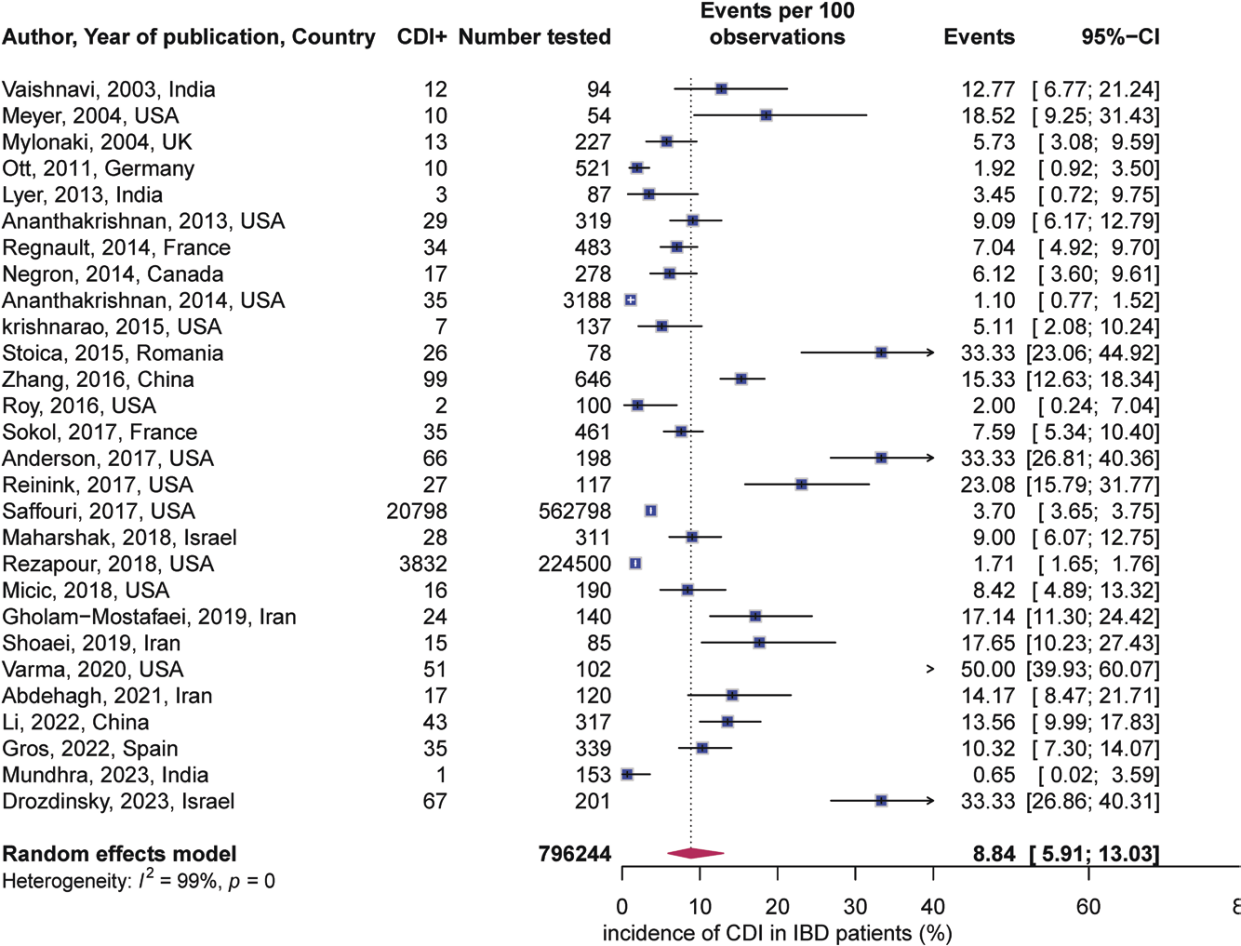
Overall incidence of CDI in IBD flare.

**Figure 3.**
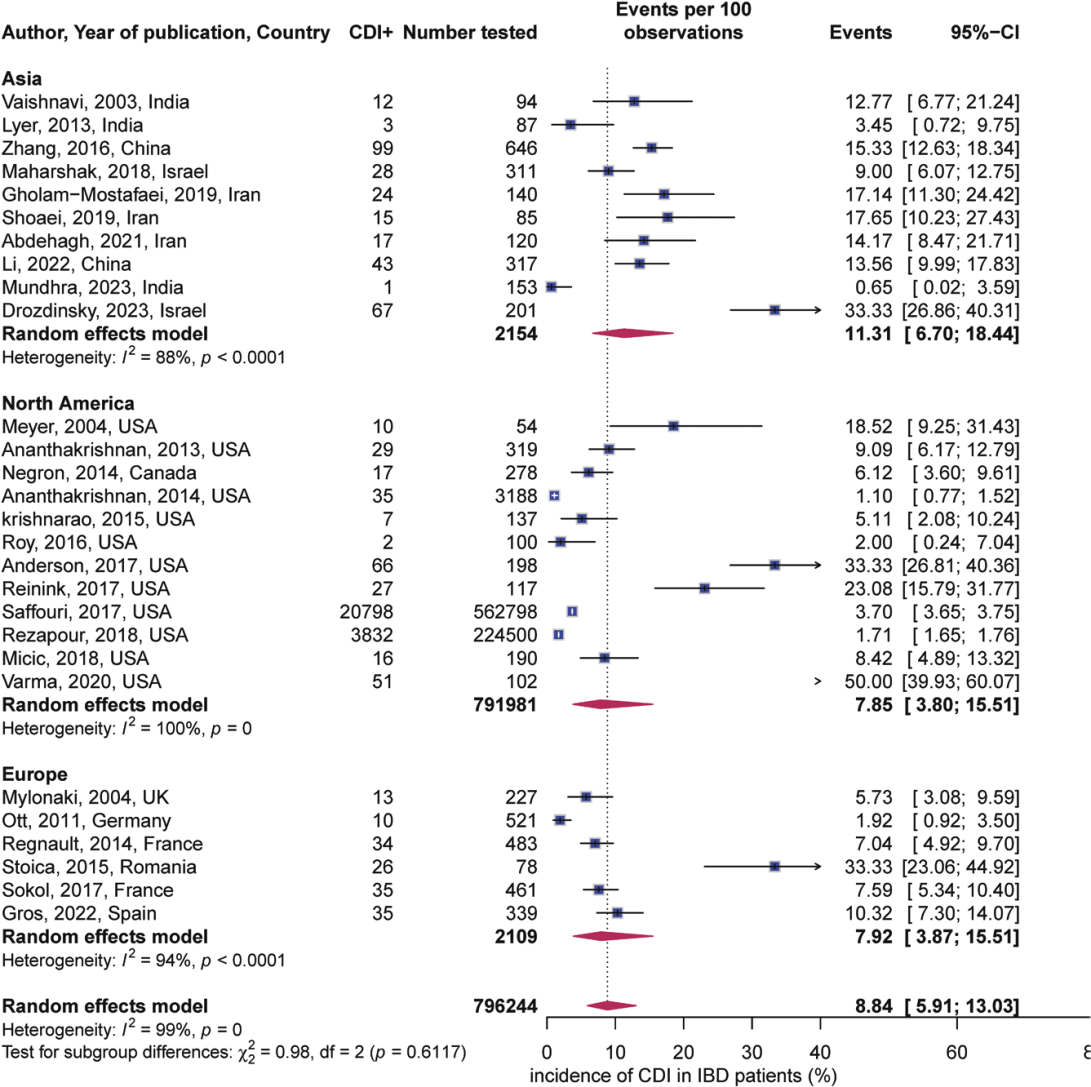
CDI incidence by geographic location stratified by continents.

Using the random-effects model, the overall CDI incidence was 8.84 (95% CI, 5.91%–13.03%) (**Figure 2**). Between study heterogeneity was high. The rate of CDI was slightly higher in Asia at 11% (95% CI, 6.7%–18.44%) compared to the North America (USA and Canada) at 7.85% (95% CI, 3.80%–15.51%) and Europe, where the incidence, was 7.92% (95% CI, 3.87%–15.51%). Test for subgroup difference, chi square = 0.98, *P* = .61 (**Figure 3**).

### Risk Factors for CDI in Inflammatory Bowel Disease

**Figure 4.**
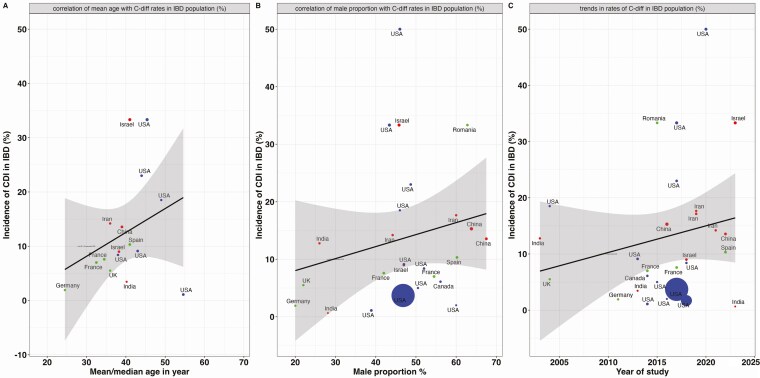
Association of age, gender, and study year with the observed incidence of CDI in IBD patients.

A univariable random-effects meta-regression model demonstrated that male (odds ratio [OR], 1.18; 95% CI, 1.00–1.40, per 5-percentage point increase), older age (OR, 1.06; 95% CI, 0.99–1.13, per each year increase from the median age) were factors associated with higher CDI incidence in IBD participants (**Figure 4**A and B) (**Table 2**). In multivariable analysis, gender and age increased the rate of CDI in IBD patient, although the association not statistically significant, male (adjusted odds ratio [aOR], 1.12; 95% CI, 0.99–1.50, per 5-percentage point increase), older age (aOR, 1.03; 95% CI, 0.97–1.1, per each year increase from the median age). CDI testing by polymerase chain reaction (PCR) compared to enzyme immunoassay was associated with higher rates of CDI (OR, 4.70; 95% CI, 01.39–15.90).

**Table 2.** Univariate meta-analysis of the risk factor for CDI in IBD flare.

Population characteristics	No of studies	OR (95% Confidence interval)	*P*-value	*R* ^2^, %
Mean age (per 1-y increase from mean of 34 y)	17	1.06 (0.99– 1.13)	.09	1.2
Proportion exposed to antibiotics (per 5-percentage point increase)	20	1.01 (0.92–1.11)	.79	0.0
Proportion PPI use (per 5-percentage point increase)	8	0.83 (0.68–1.01)	.06	26.3
Proportion male (per 5-percentage point increase)	25	1.18 (1.00 –1.40)	.056	49
White (per 5- percentage point increase) US and Canada studies)	5	0.87 (0.60–1.27)	.36	0.0
Year of study (per 5- y increase)	28	1.19 (0.78–1.81)	.45	0.0
Proportion ulcerative colitis (per 5-percentage point increase)	28	1.02 (0.95–1.10)	.61	0.0
**Method of test used**			.04	17
PCR	4	4.70 (01.39–15.90)		
Multiple methods	3	0.89 (0.36 –2.19)		
EIA	16	Reference (EIA)		

Prior exposure to antibiotics, year of study, and UC were each associated with higher rates of CDI, although the associations did not reach statistical significance. No association was observed between proton pump inhibitor use, race, and CDI infection in the IBD population. Temporal trend analysis indicated that the CDI incidence rate has been on the slow rise for the past 2 decades (*R*^2^ = 2%; *P* = .026 for temporal trend, **Figure 4C**).

### Outcomes of CDI in Inflammatory Bowel Disease

We characterized the association between length of hospital stay with CDI in the IBD population. Due to fewer studies reporting mortality, this was not qualitatively analyzed. No association was observed between LOS and CDI infection (**Table 3**).

**Table 3.** Association of CDI incidence and outcomes in IBD flare population.

Outcomes	No of studies	OR (95% ci)	p*P*-value	*R* ^2^, %
Hospital length of stay (per 1-day increase)	5	0.95 (0.66–1.39)	.81	.00

### Sensitivity Analyses

**Figure 5.**
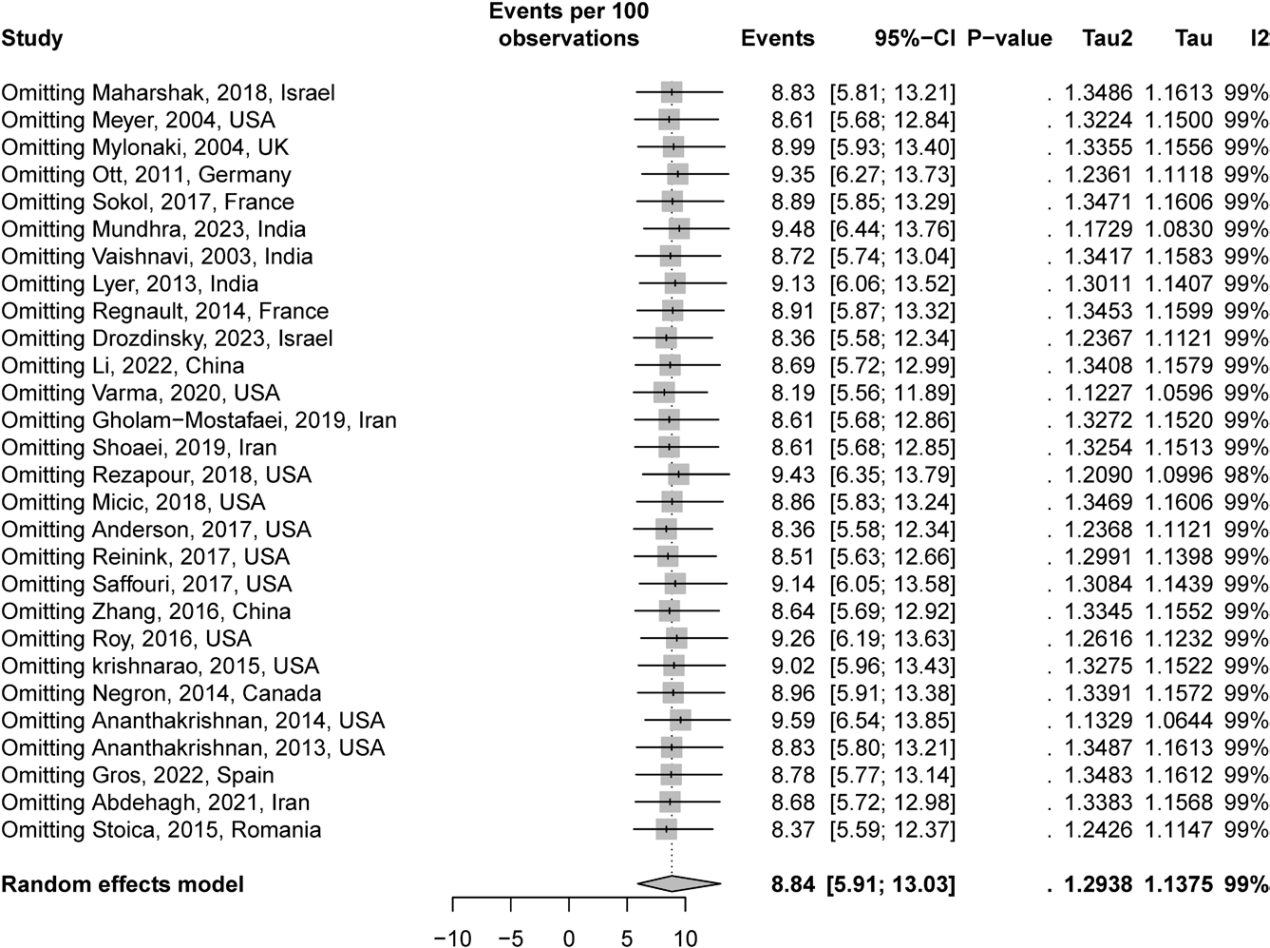
Influence and outlier sensitivity analyses. No study was an outlier or substantially influenced the pooled estimates.

Influence and outlier sensitivity analyses were performed to determine if a particular study influenced the observed incidence of CDI in patients with IBD. In this analysis, one study was omitted and replaced one study at a time (leave-one-out method) from the meta-analysis, and we calculated the pooled data for the remaining studies. The pooled estimate remained close to the observed overall pooled estimate, indicating that no individual study had a considerable influence on the pooled estimate. The pooled point estimate ranged from 8.19% to 9.59 % (**Figure 5**).

**Figure 6.**
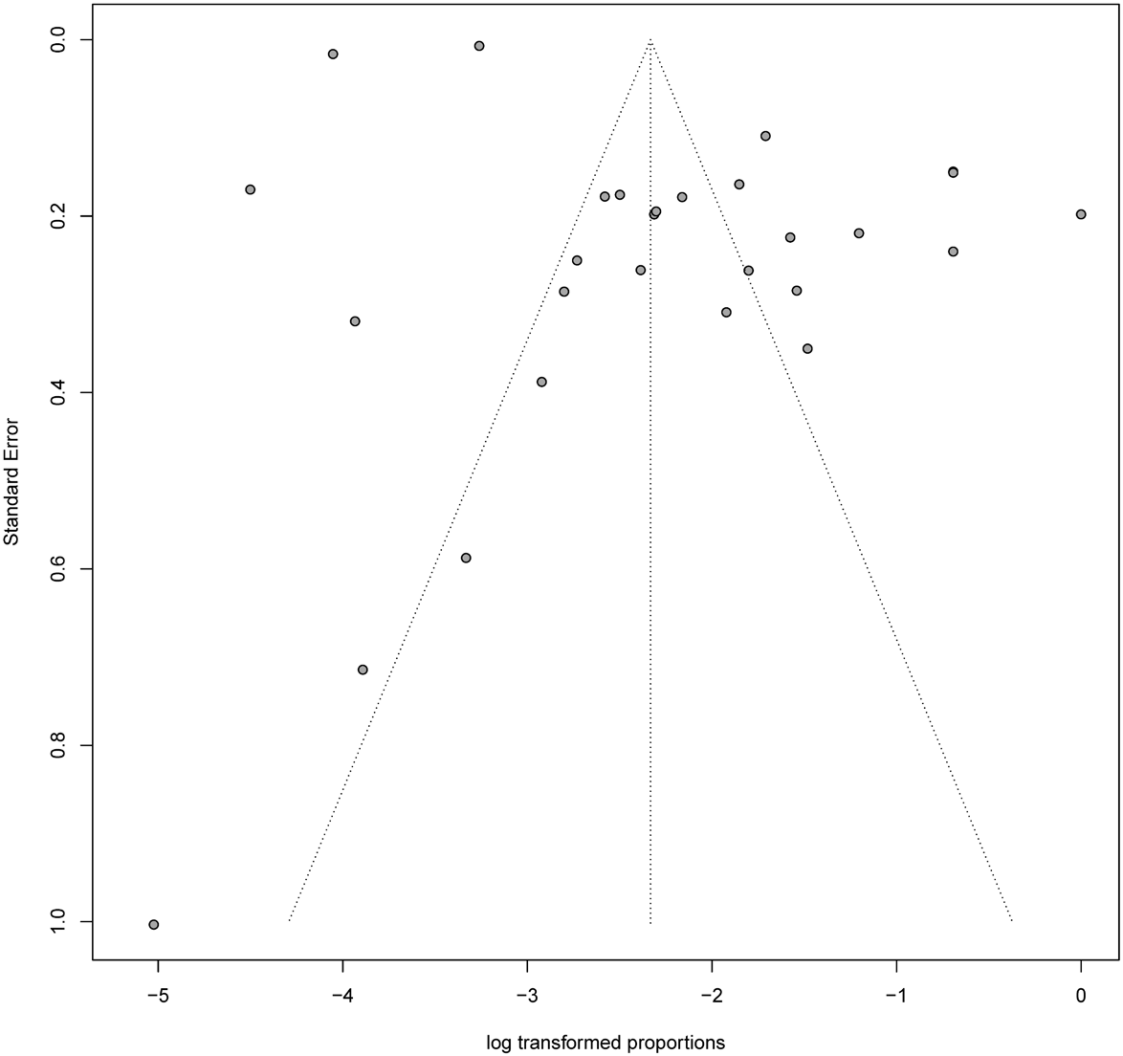
Funnel plot demonstrating the distribution of effect estimates a function of sample size of the study.

**Figure 7:**
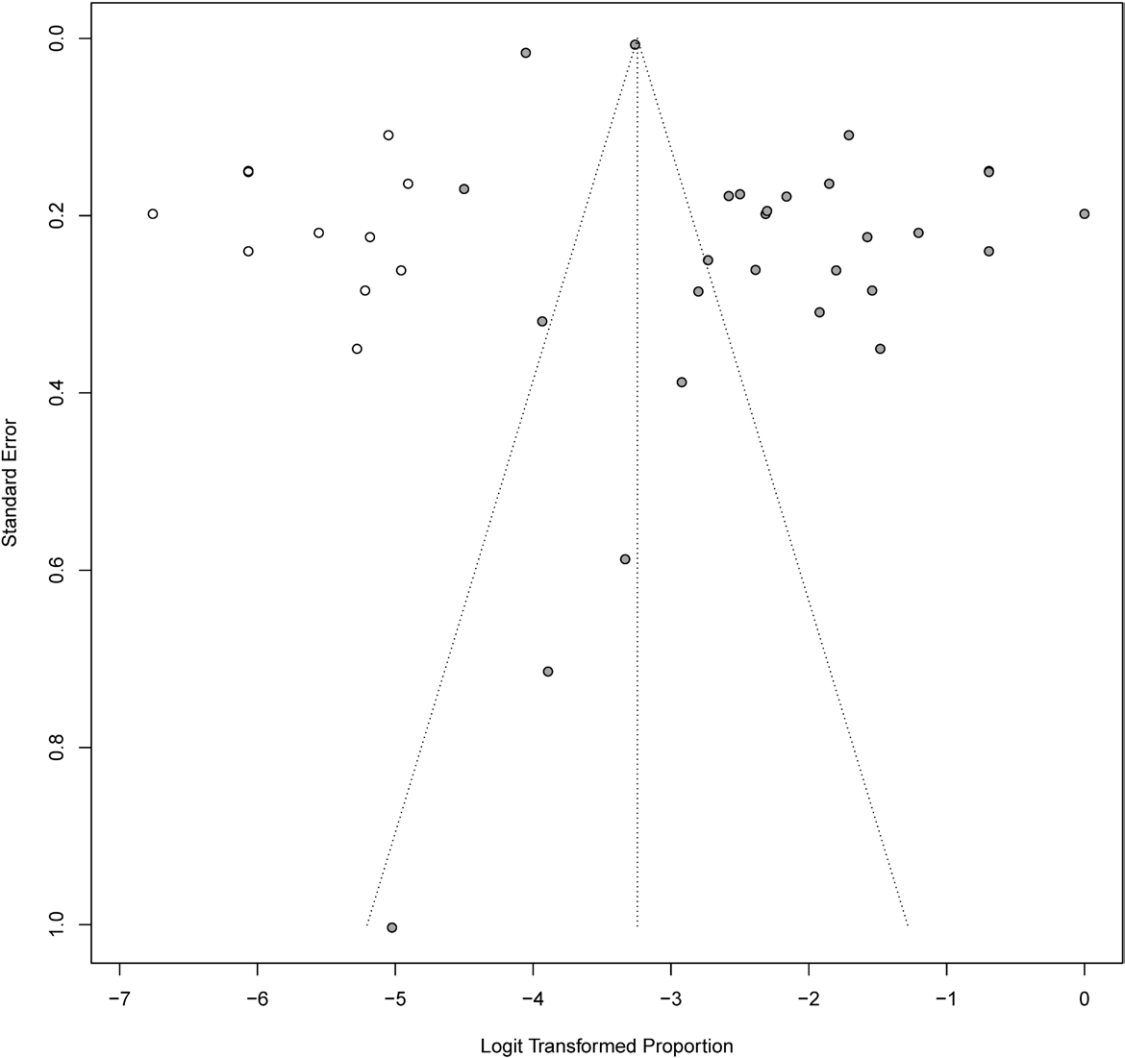
Duval and Tweedie trim and fill analysis: 12 additional studies were added balance the funnel plots.

Asymmetrical inverted funnel plot suggested the presence of publication bias (**Figure 6**). Similarly, both Begg rank correlation test (*z* = -3.12; *P* = 0.02) and the Egger linear regression test (*t* = 2.23; *df* = 26; *P* = .03) indicated publication bias. Duval and Tweedie trim and fill analysis was conducted to adjust for the potential small-study publication bias. Analyses suggested that the adjusted effect estimates would fall from 2.17% to 6.43% and 11 additional studies were added to the funnel plots (**Figure 7**).

## Discussion

In this systematic literature review and meta-analysis of nearly 800 000 patients with inflammatory bowel disease, the global incidence rate of CDI in the IBD population was 9%. Older age and male population were associated with the increased rates of CDI in inflammatory bowel disease participants. Although prior antibiotic exposure and the use of PCR to diagnose CDI were associated with higher rates of *C. diff*, the association did not reach statistical significance. Overall, the infection rate is slightly increasing over time.

While CDI incidence are reportedly higher in IBD patients compared to the general population,^24^ our literature search did not identify any study specifically reporting the global incidence of CDI in IBD. Our findings provide valuable data that can guide clinical decisions on *C. diff* testing in patients admitted with IBD. The CDI rate was higher in the Asian subcontinent at 11% and in North America (USA and Canada) at 10% when compared to Europe 8%. This contrasts with earlier reviews that reported a higher incidence of CDI in North America and Europe and a lower incidence in Asia,^1^ attributing these differences to more virulent strains NAP1/ribotype 027 and 078 found in the USA and Europe.^25^ This study included a larger sample size and data from 11 countries (4 in Asia and 5 in Europe) which may explain these unique findings. Additionally, differences in testing protocols such as emphasis on free toxin detection in stool in Europe, may also have contributed to the variation observed in results.^26^

As age increased, the odds of CDI in IBD patients also increased (OR, 1.07; 95% CI, 1.0–1.15, per one year increase from median age) was observed and this is consistent with trends in the general population.^27,28^ The heightened risk may be due to the decreased efficacy of the innate immune system and changes in intestinal microbiota with older age.^29^ These factors increase the susceptibility of elderly patient population to CDI compared to the young population. Additionally, male gender was associated with higher odds of CDI (odds ratio [OR], 1.25; 95% CI, 1.05–1.50). The mechanism behind this increased risk is not fully understood. Previous studies have yielded conflicting conclusions on the role of gender in CDI in this cohort of patients. Balram et al. found no significant association between gender and CDI in IBD,^30^ whereas another study also reported an increased risk of CDI in male patients with IBD, specifically those with ileal pouches.^31^ However, in the general population, CDI tends to be associated with the female gender due to multiple factors such as hormonal differences, higher healthcare exposure and pregnancy.^27,32^ Further research is needed to clarify the role of gender in CDI among IBD patients.

Furthermore, this study revealed that while PCR-based diagnosis of CDI and prior antibiotic exposure were associated with higher rates of CDI, these associations did not reach statistical significance. Previous studies that established a link between antibiotic use and CDI typically followed subjects for up to 60 days post-discharge.^33^ We could not evaluate the duration of antibiotic use due to limitations in the availability of data, which may have influenced our findings. There was no association between proton pump inhibitor (PPI) usage and CDI in the IBD population. This finding is consistent with earlier studies, which also found no association.^30^ PPIs are known to alter the gut microbiota, induce changes in colonocyte gene expression, and also enhance the probability of *C. diff* spores getting to the colon through decreased gastric acid suppression, thereby increasing CDI risk.^28,34,35^ The already altered gut microbiome of IBD patients may negate any additional CDI risk posed by PPI use. Additionally, temporal trend analysis revealed an increasing incidence of CDI over the past 2 decades; this is in alignment with results from other studies.^36^

*C. diff* frequently colonizes the large bowel of both humans and other mammals.^1^ There are both toxigenic and non-toxigenic strains, and a CDI occurs depending on host factors such as immune system response and resistance to colonization.^37^ The presence of about 4,000 different commensal bacteria in the large intestines controls the toxigenic *C. diff* population through competition for gut wall attachment and essential nutrients thereby decreasing the likelihood of CDI.^38,39^

Patients with IBD are at an increased risk of CDI due to several factors. Firstly, intestinal dysbiosis in IBD alters the natural balance between commensal bacteria and *C. diff*, leading to the optimal conditions for *C. diff* proliferation.^40^ Additionally, IBD patients are frequently hospitalized during flare-ups and are more likely to be exposed to antibiotics compared to the general population.^41–43^ Colonization at the time of admission increases the risk of CDI 6-fold.^42^ Antibiotics decrease the population of essential bacteria such as *Bacteroides* and *Firmicutes* phylum increasing the risk of CDI.^26^ Immunosuppressive medications including corticosteroids and Disease Modifying Anti Rheumatic Drugs (DMARDS) used in IBD treatment can impair the host immune response which increases the likelihood of CDI in colonized patients.^28^

This study has significant clinical and public health implications. Some experts suggest that CDI in IBD not only indicates a challenging condition but also serves as a marker for disease activity.^28^ Therefore, the high incidence of CDI observed in this study underscores the critical need for testing for *C. diff* during IBD flares. This practice is also recommended by the American College of Gastroenterology (ACG) due to the difficulty in clinically distinguishing between an IBD flare and CDI.^28^

IBD patients, who already face multiple healthcare exposures, are at an increased risk of poor outcomes when diagnosed with CDI. These include prolonged hospitalization, healthcare utilization, and higher cost of care. Additionally, CDI can lead to IBD treatment failure, necessitating surgical intervention and colectomy, and often requires intensification of IBD treatment which comes with its own set of adverse effects.^30,44^ Timely diagnosis and treatment of CDI in IBD are essential in improving patient outcomes and mitigating these complications.

Our study has a few limitations. Firstly, despite our systematic literature search, we encountered publication bias, as evidenced by the asymmetric funnel. Both Begg’s rank correlation and Egger’s linear regression also confirmed the presence of publication bias. Additionally, we did not investigate the duration of antibiotic use, which is a major factor that has been associated with CDI.

## Conclusion

In this systematic literature review and meta-analysis, the incidence of CDI in IBD flares is 9%. Significant factors included male gender and increasing age. There was no association between PPI use and risk of CDI in IBD patients. These results underscore the importance of testing for CDI during IBD flares and initiating treatment when necessary to reduce morbidity and mortality. Future research should focus on identifying subgroups with a higher burden of CDI and understanding their outcomes.

## Data Availability

The data analyzed in this study is available in the referenced studies in this article.
